# Integrating photoacoustic tomography into a multimodal automated breast ultrasound scanner

**DOI:** 10.1117/1.JBO.25.11.116010

**Published:** 2020-11-19

**Authors:** Corey Kelly, Amir Refaee, Septimiu E. Salcudean

**Affiliations:** University of British Columbia, Department of Electrical and Computer Engineering, Vancouver, British Columbia, Canada

**Keywords:** photoacoustics, tomography, optical design, ultrasonics, medical imaging, image reconstruction

## Abstract

**Significance:** Photoacoustic tomography (PAT) is a promising emergent modality for the screening and staging of breast cancer. To minimize barriers to clinical translation, it is common to develop PAT systems based upon existing ultrasound hardware, which can entail significant design challenges in terms of light delivery. This often results in inherently non-uniform fluence within the tissue and should be accounted for during image reconstruction.

**Aim:** We aim to integrate PAT into an automated breast ultrasound scanner with minimal change to the existing system.

**Approach:** We designed and implemented an illuminator that directs spatially non-uniform light to the tissue near the acquisition plane of the imaging array. We developed a graphics processing unit-accelerated reconstruction method, which accounts for this illumination geometry by modeling the structure of the light in the sample. We quantified the performance of this system using a custom, modular photoacoustic phantom and graphite rods embedded in chicken breast tissue.

**Results:** Our illuminator provides a fluence of $2.5\;\mathrm{mJ}\;{\mathrm{cm}}^{-2}$ at the tissue surface, which was sufficient to attain a signal-to-noise ratio (SNR) of 8 dB at 2 cm in chicken breast tissue and image 0.25-mm features at depths of up to 3 cm in a medium with moderate optical scattering. Our reconstruction scheme is 200× faster than a CPU implementation; it provides a 25% increase in SNR at 2 cm in chicken breast tissue and lowers image error by an average of 31% at imaging depths >1.5  cm compared with a method that does not account for the inhomogeneity of the illumination or the transducer directivity.

**Conclusions:** A fan-shaped illumination geometry is feasible for PAT; however, it is important to account for non-uniform fluence in illumination scenarios such as this. Future work will focus on increasing fluence and further optimizing the ultrasound hardware to improve SNR and overall image quality.

## 1 Introduction

Ultrasound has long served as a supplement to mammography for peripheral sites, as a “second-look” modality, especially for women with dense breasts, who are at higher risk of developing breast cancer and for whom the gold standard of mammography falls short.^1^^–^^3^ Multimodal imaging, and in particular, photoacoustic tomography (PAT), of the breast has emerged as a promising clinical tool for the detection,^4^^,^^5^ staging,^6^^,^^7^ and pre-operative margin assessment^8^ of breast malignancies.

The SonixEmbrace automated breast ultrasound scanner (ABUS—Ultrasonix Medical Corporation, Richmond, British Columbia, Canada) consists of a large, concave ultrasound transducer embedded in a spherical dome. The patient lies prone on a padded platform with one breast lying against a gel-based custom coupling pad within the imaging dome. A motor rotates the dome through 360°, acquiring B-mode images at 0.5° intervals, which are then interpolated to produce volumetric ultrasound. The SonixEmbrace system has several advantages outlined by Azar et al,^9^ including high lateral resolution due to the concave transducer array design, a fixed scanning volume that facilitates registration to other imaging modalities such as magnetic resonance imaging (MRI), and rapid volumetric acquisition time of 2 min. The breast does not move nor is it deformed during the acquisition, and therefore, its volumetric image can be re-sliced for analysis and diagnosis. Thus operator-dependence in image acquisition is effectively removed, and the diagnosis by a qualified radiologist could be facilitated.

This rigid volume scanning scheme and tomographic geometry of the SonixEmbrace provide an excellent platform for multimodal imaging. We have previously demonstrated simultaneous acquisition of tomographic ultrasound, elasticity, Doppler flow, and photoacoustic imaging in a tissue-mimicking phantom and presented our preliminary results.^10^ Integrating PAT into this scanning geometry without sacrificing quality of the other modalities presents challenges in hardware and optics design and in data reconstruction.

The state of the art in breast PAT was recently summarized by Manohar and Dantuma,^11^ and we refer the reader to this review for a detailed description of common imaging and illumination geometries currently in use. Such systems are carefully designed from the ground up to balance the density of detection elements against the ease of delivering illumination to the sample. Illuminating the entire surface of the tissue with a collimated or divergent beam is often preferable, and this geometry has seen widespread use for the imaging of small animals^12^^,^^13^ and human extremities.^14^ This type of illumination has the advantage of providing a relatively uniform fluence at the tissue surface, such that the fluence within the imaging volume can be either assumed constant or accounted for with a depth-based attenuation model.^15^ In the case of the SonixEmbrace, where the ultrasound hardware is a given and we wish to retrofit the illumination, we are more limited in our options. In particular, for reasons elaborated upon in Sec. 2.1, a collimated beam illuminating the entire surface of the breast is not possible.

Taking inspiration from the ever-growing body of work integrating illumination hardware into freehand ultrasound systems,^15^^–^^17^ we explored diffusive, beam-shaping optics that can be placed near the transducer to provide fluence in the acoustic acquisition plane. In 2018, we presented preliminary results showing feasibility of a fan-shaped diffuse beam for this purpose.^18^ This illumination approach remains novel among tomographic breast PAT systems, and in the present study, we provide a complete characterization of both our hardware and software and a new graphics processing unit (GPU)-accelerated reconstruction scheme. At the same conference, Oraevsky et al.^19^ presented the laser optoacoustic ultrasonic imaging system assembly (LOUISA)-3D system, which is superficially similar to our own in its use of a curved transducer array and a moving illuminator, but differs in several significant ways. Most relevant is that their system was designed to include a transparent imaging dome allowing the illuminator to be placed nearer to the tissue and to illuminate a larger area with a single laser pulse. This illumination system also moves independently of the transducer array, whereas these are coupled in our system. The authors describe the illuminator itself as an arc-shaped “paddle” with multiple fiber optic segments, which is distinct from our simpler single-fiber solution.

We also note that our design is distinct from existing systems that employ diode light bars or rectangular shaping diffusive elements^17^^,^^20^ in that the light diverges significantly in the space between the optics and the tissue, resulting in inherently non-uniform fluence at the tissue boundary. While these optics offer flexibility in terms of the light delivery, this spatial non-uniformity of the fluence must be accounted for during data reconstruction.^21^^–^^23^

Spatial fluence modeling is commonly used in photoacoustic simulations to provide an accurate ground truth for experimental validation,^20^^,^^22^ and this is usually accomplished via a Monte Carlo simulation of the radiative transfer equation (RTE). It is also often necessary in quantitative photoacoustic tomography to fully model the fluence such that the optical parameters of the tissue can be recovered absolutely, for example, in the recent work of Hänninen et al.^24^ In motivating their work, the authors highlight that a significant shortcoming of the standard Monte Carlo method is the necessary computation time, which can be prohibitive for large tomographic problems such as ours. This is further compounded in the case of non-stationary illumination since the fluence must be computed separately for each tomographic acquisition angle. In the present study, we employ a first-order approximation to the RTE to lower this computational burden.

We note the unique recent work of Park et al.^25^ which estimates and accounts for non-uniform illumination and attenuation as a part of the image reconstruction. Our work differs from this approach in that we do not need to compute the location of the breast surface and that we do not assume constant fluence at the sample surface.

In this study, we present a novel, fan-shaped illuminator for PAT of the breast, and we characterize and quantify the performance of this illumination system in terms of the fluence that it can deliver to the tissue surface. We also explore the use of an approximate, model-based method for deconvolving spatially-varying illumination from measured photoacoustic signals and quantify the benefits of using such a method. To our knowledge, this study constitutes the first attempt to integrate PAT into a pre-existing multimodal ABUS system. By attempting to add PAT to this system with minimal changes to the existing design, we have made compromises relative to the current state of the art. In particular, our transducer is a conventional array that is designed primarily for B-mode imaging. Our approach of using diffusive optics and modeling the illumination is general enough to be used in other photoacoustic imaging applications, especially those attempting to minimize cost,^20^ image disease sites that are difficult to illuminate by conventional means such as the prostate,^26^^,^^27^ and guide minimally invasive surgical procedures in which space constraints often lead to inherently non-uniform illumination.^28^

## 2 Methods

### 2.1 SonixEmbrace ABUS

**Fig. 1:**
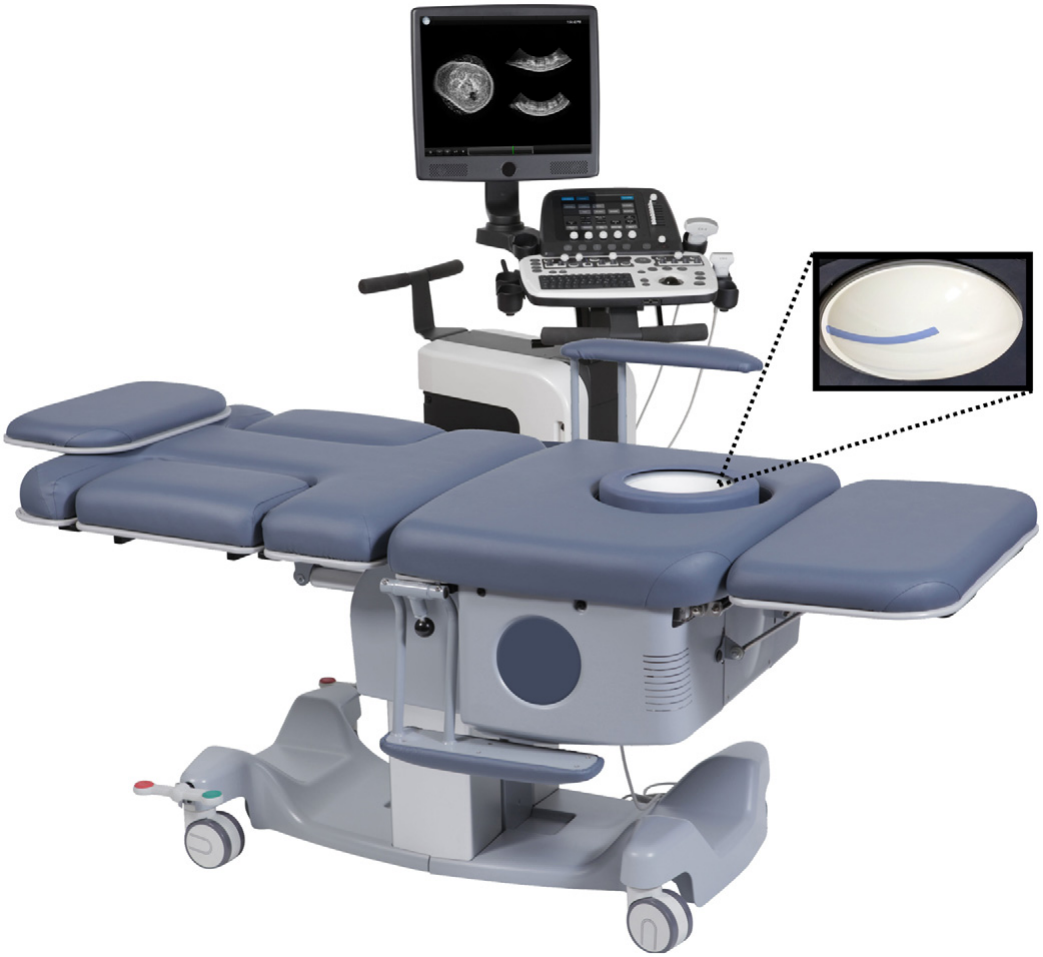
The SonixEmbrace ABUS system, consisting of the ultrasound PC and scanning platform. Inset shows a top-down view of the transducer dome, with the blue transducer array visible.

**Fig. 2:**
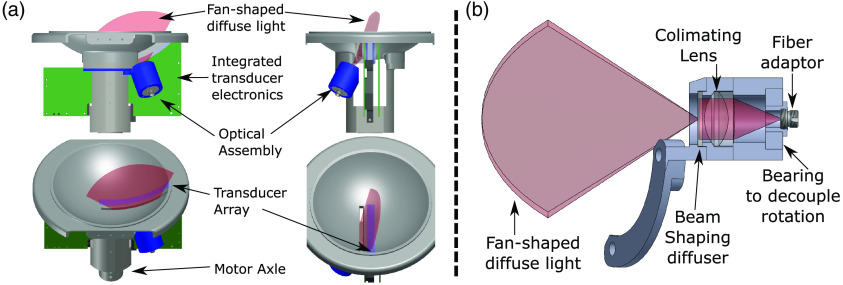
Schematic diagrams showing (a) the position of the ABUS illuminator and the electromechanical components beneath the ABUS imaging dome and (b) the illuminator, with the cutaway view revealing the optical elements.

The SonixEmbrace transducer array consists of 384 elements on a 11.5-cm long circular arc with a curvature of −12  cm. It has a center frequency of 10 MHz and 90% bandwidth. The transducer dome has a radius of curvature of 12 cm and a diameter of 18 cm. A photograph of the dome is shown in Fig. 1. This large transducer requires multiplexing (mux) hardware that occupies a significant amount of space directly behind the transducer array under the dome (see Fig. 2), limiting conventional illumination options for breast PAT such as a collimated, expanded beam.^19^^,^^29^ In further contrast to these systems, the SonixEmbrace uses a thin (∼5  mm) gel pad for acoustic coupling, as opposed to a flexible membrane or liquid-filled chamber. This means that there is little provision for any divergent optics to expand to cover a significant area of the tissue surface. We explored the possibility of using diode-based illumination, as the smaller footprint of such systems could potentially place the illumination closer to the surface. These systems would provide lower pulse power, so we chose to use a laser-based system in the interest of maximizing fluence and, therefore, the attainable penetration depth. We can also use an optical parametric oscillator (OPO) to perform multi-spectral PAT without changes to the output optics, whereas a diode system would require one illuminator per wavelength.

The mechanical constraints imposed by the configuration of the ABUS electronics and motor led us to consider a narrow, fan-shaped beam that could fit between the multiplexer boards and the motor axle. We 3D printed an identical transducer dome in poly(lactic acid) (PLA; Afinia, Chanhassen, Minnesota), which has a polycarbonate optical window epoxied parallel to the transducer, as shown in Fig. 2.

### 2.2 Laser and Optics

Our illumination system consists of a Continuum Surelite II LASER (Continuum Inc, Santa Clara, California) coupled to an OPO from the same manufacturer to control the output wavelength in the range of 675 to 2500 nm. We use a 80/20 beam splitter (68-376, Edmund Optics, Barrington, New Jersey) to direct 20% of the OPO output to a USB power meter (EnergyMax J-50MB-YAG – Coherent Inc., Santa Clara, California), allowing us to measure per-pulse energy while imaging. The remainder of the beam has a diameter of 9.5 mm, and is subsequently homogenized using a cross cylindrical lens array (Nr.18-00142, SUSS MicroOptics, Hauterive, Switzerland) and coupled by means of a plano-convex spherical lens (LA1608, Thorlabs, Newton) into a 1-mm diameter silica core optical fiber. Our beam homogenization and coupling system are described in further detail in Ref. 30. This system provides 5-ns pulses at 700 nm with a per-pulse energy at the output end of the fiber exceeding 50 mJ.

At the output end of the fiber is an optical assembly contained in a 3D-printed housing to maintain alignment and direct the diffuse output toward the optical window in the ABUS dome. The fiber is connected to the optical assembly by means of a ferrule connector (FC) fiber bulkhead connector (Thorlabs, Newton, New Jersey) and a bearing to decouple the fiber from the dome rotation. The fiber output first passes through an achromatic collimating doublet (65-438, Edmund Optics, Barrington, New Jersey) and is then incident upon an engineered “line diffuser” (EDL-100×0.4, RPC Photonics, Rochester, New York), which shapes the collimated beam into a fan. This fan has a 90° divergence along one axis and a 0.24° divergence along the other. This shape permits the beam to pass through the optical window, while illuminating its entire 11.5-cm length. A cross-sectional view of this assembly is shown in Fig. 2.

### 2.3 Data Acquisition and Pre-Processing

Radio frequency (RF) data from the ABUS transducer were acquired using a SonixDAQ module (BK Medical). The SonixDAQ has a sampling rate at 40 MHz, a 12-bit resolution, and a −10-dB sensitivity in the range 2 to 20 MHz. Since the SonixDAQ can only acquire 128 channels at a time, each plane acquisition requires three acquisitions to cover all of the transducer elements. Control of the ABUS motor, and all triggering and synchronization of the optics and DAQ acquisition was accomplished using a custom Arduino-based circuit controlled over USB from a Windows PC.

**Fig. 3:**
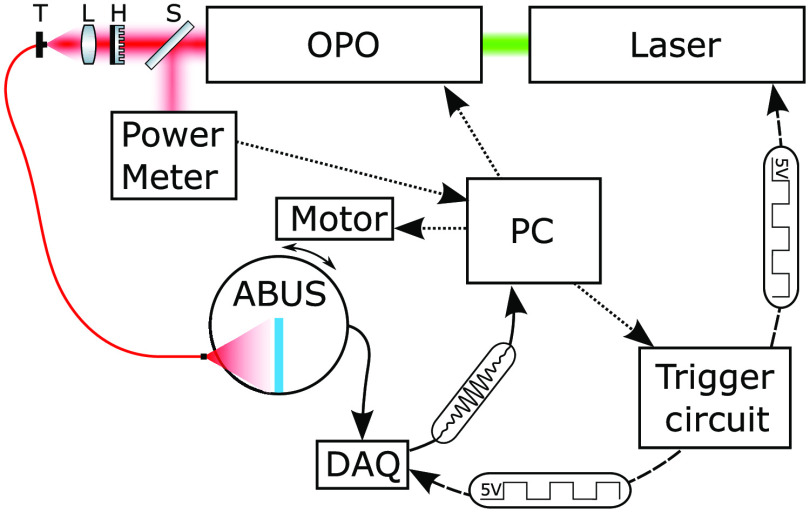
The experimental setup used in this study. Optical elements T, L, H, and S are a translation stage, lens, beam homogenizer, and beam splitter, respectively. Dotted lines represent serial communications, and dashed lines are synchronization triggers.

This circuit allows flashlamp triggers to continually be sent to the laser at its pulse repetition rate of 10 Hz, while illumination pulses (via q-switch triggers) can be requested via hardware or software triggers. We have found that this “steady-state” operation decreases inter-pulse energy variability. For the pulse energies that we employ in this study, this variability is on the order of 10% to 15%. To further minimize the effect of this variability, we use the power meter data to normalize each 128-element acquisition by the relative pulse energy. A diagram summarizing the system configuration is shown in Fig. 3.

Several steps are needed to pre-process the RF data prior to photoacoustic reconstruction. First, each A-line is low-pass filtered to remove high-frequency components outside the working range of the transducer. This was performed in software using a fifth-order Butterworth filter with a cutoff at 14.5 MHz. We retain the low-frequency components at this stage as they are essential for the following processing step: the application of singular value decomposition (SVD) denoising to remove cross-channel noise bands.^31^ To perform this denoising, we compute the SVD of a 128×2080 matrix in which each row corresponds to the RF data for one transducer element. In this representation, this band-shaped noise dominates the first few singular value components. We can isolate these components and revert to the original representation, producing a matrix consisting of only the noise. This can then be subtracted from the original data, resulting in the denoised matrix. Since these noise bands are stochastic, this decomposition and denoising must be performed for each 128-element acquisition. Using the GPU implementation of SVD available in the scikit-cuda Python package,^32^ this denoising takes only 100 ms for each 128×2080 array. Any low-frequency components outside the working range of the transducer will be removed by a ramp filter described in Eq. (3).

We explored several other denoising approaches including template matching, directional filtering^33^ (both to the radio-frequency and image data), and temporal averaging (i.e., acquiring multiple frames of RF data per transducer position). Of these, temporal averaging was most successful at reducing the prominence of the noise bands, although it comes at a cost of increasing acquisition time. Our 10-Hz laser system and SonixDAQ create significant overhead for each additional acquisition, especially since the ABUS array requires three 128-element acquisitions per transducer position. For a volumetric scan of 200 angular positions, acquiring a single frame per position resulted in a 20-min scan time, whereas acquiring 10 frames per position increased the scan time to well over 1 h.

The other denoising approaches were either very sensitive to input parameters or unacceptably detrimental to the image quality. We have found that the SVD-based approach works on a wide variety of data with the same input parameters, removes the noise bands with minimal effect on the photoacoustic signals, and is computationally efficient to apply to the data. We have included an example comparing the effectiveness of directional filtering and SVD-based denoising in the Supplementary Materials.

### 2.4 Reconstruction

When a laser pulse with spatial energy distribution Ψ(r,t) at time t and positions r is incident upon a sample with coefficient of thermal expansion β, heat capacity $C_{v}$, acoustic speed $v_{s}$, and optical absorption coefficient $\mu_{a}(r)$, an acoustic wave is generated with initial pressure distribution

$$p_{0}(r,t)=\frac{v_{s}^{2}\beta }{C_{v}}\Psi (r,t)\mu_{a}(r).$$ (1)

Assuming the medium properties β, $C_{v}$, and $v_{s}$ are spatially homogeneous and the laser pulse is temporally short, Ψ(r,t)=Ψ(r)δ(t), we have

$$p_{0}(r)\propto \Psi (r)\mu_{A}(r),$$ (2)

at time t=0, when the laser is incident upon the sample. The goal of PAT is to reconstruct $\mu_{a}(r)$ for the set of positions r, based on a set of time-varying pressure values $p_{D}(r_{D},t)$ measured at discrete detector positions $r_{D}$. This involves first inverting the measurements $p_{D}(r_{D},t)$ to find the initial pressure distribution $p_{0}(r)$. For a spherical scanning geometry, Xu et al.^34^ expressed this inversion in a back-projection form as

$$p_{0}(r)=\frac{2}{\Omega_{0}}\int_{\Omega_{0}}d\Omega_{0}{\left[p_{D}(r_{D},t)-t\frac{\partial p_{D}(r_{D},t)}{\partial t}\right]}_{t=\frac{\left|r-r_{D}\right|}{v_{s}}},$$ (3)

where $\Omega_{0}$ is the solid angle of the detection surface containing the points $r_{D}$ and $d\Omega_{0}$ is the solid angle of the surface element at a location $r_{D}$ relative to a sample point r. While this form is exact only in the case in which this surface completely encloses the sample ($\Omega_{0}=4\pi$), the $\frac{d\Omega_{0}}{\Omega_{0}}$ term serves as a weight that will somewhat mitigate the effects of the well-known partial view problem.^34^

Finding $p_{0}$ in Eq. (3) is typically accomplished using back-projection algorithms such as delay-and-sum and is a well-studied problem.^35^ More sophisticated reconstruction algorithms take into account the fact that the measured pressure data are not exactly equal to the actual pressure incident on the detector due to factors such as the electromechanical and spatial impulse responses (SIRs) of the detector.^35^^,^^36^ We can generalize the measured pressure data as

$$p_{D}(r_{D},r,t)=E(t)*S(r_{D},r,t)*p(r_{D},t),$$ (4)

where $p(r_{D},t)$ is the actual time-varying pressure incident on the detector at $r_{D}$, $S(r_{D},r,t)$ is the SIR of the detector at $r_{D}$ relative to measurement point r, E(t) is the electrical impulse response (EIR), and * denotes linear convolution with respect to time. Here we consider only the spatial sensitivity of the transducer, i.e. we assume a flat frequency response, giving

$$E(t)=\delta (t),\;p_{D}(r_{D},r,t)=S(r_{D},r,t)*p(r_{D},t).$$ (5)

Since the SonixEmbrace transducer was designed for ultrasound imaging, it includes an acoustic lens for beam focusing, and as such it would be a poor assumption to fully neglect the SIR, as is common in many PAT reconstruction schemes. It is known, however, that accounting for the SIR is computationally difficult.^35^^,^^36^ In an attempt to strike a balance, we separate the SIR into a spatial portion that describes the directional sensitivity and a temporal portion that describes the averaging effect due to the finite size of the transducer element

$$S(r_{D},r,t)=S_{0}(r_{D},r)S_{1}(t),$$ (6)

and proceed under the assumption that $S_{1}(t)=\delta (t)$. This implies that the elements are point-like, but still have a non-uniform directional response

$$p_{D}(r_{D},r,t)=S_{0}(r_{D},r)p(r_{D},t).$$ (7)

Under this assumption, $S_{0}(r_{D},r)$ has no time dependence and as such does not depend on $\left|r-r_{D}\right|$ when substituted into Eq. (3). This assumption also implies that the transducer has a flat frequency response. In the coordinate system of one transducer, we define $\theta_{L}$ and $\theta_{E}$ as the angles between the element normal (the axial direction) and the sample point in the lateral (in-plane) and elevational (out-of-plane) directions, respectively, such that

$$p_{D}(r_{D},r,t)=S_{0}(\theta_{L},\theta_{E})p(r_{D},t).$$ (8)

Since we are already computing the total angle between the element normal and the sample point to calculate the solid angle element $d\Omega_{0}$ in Eq. (3), $S_{0}(\theta_{L},\theta_{E})$ does not appreciably change the computational effort required. Since $\theta_{L}$ and $\theta_{E}$ must be computed for every pair of $r_{D}$ and r ($∼10^{5}\cdot 10^{7}=10^{12}$), this approach lends itself well to parallelization using GPUs.

#### 2.4.1 Non-uniform illumination

If the illumination source is spatially uniform within the sample, i.e., Ψ(r)=Ψ, Eq. (2) implies that $\mu_{a}(r)$ is directly proportional to the initial pressure $p_{0}(r)$. If the illumination is non-uniform, we must deconvolve these two spatial distributions. Since strong light attenuation in tissue limits imaging depth, it is common in PAT to apply an exponential weighting to the reconstructed $p_{0}(r)$ to enhance signals further from the surface. Our illumination is not collimated (uniform) at the tissue surface, but it can still be accounted for in a similar manner.

With sufficient geometric constraints, we model Ψ(r) as a solution of the RTE for homogeneous medium with temporally short pulses.^37^ For an illumination source given by $r_{i}$, the optical fluence at a point r will be a function of the distance $r=|r-r_{i}|$ from the source:

$$\Psi (r-r_{i})=\frac{1}{4\pi Dr}\;\exp(-\mu_{\mathrm{eff}}r),$$ (9)

where the effective attenuation coefficient $\mu_{\mathrm{eff}}$ and the photon diffusion coefficient D are defined as

$$\mu_{\mathrm{eff}}=\sqrt{\frac{\mu_{a0}}{D}}\;D=\frac{1}{3(\mu_{a0}+\mu_{s^{\prime }})}\;\mu_{s^{\prime }}=\mu_{s}(1-g),$$ (10)

with $\mu_{a0}$ and $\mu_{s}$ being the bulk absorptive and scattering attenuation coefficients, respectively, and g being the tissue anisotropy. Equation (9) holds in the diffusion limit, when $\mu_{a0}\ll \mu_{s}$. For human breast tissue at 700 nm, $\mu_{s}=10.0\;{\mathrm{cm}}^{-1}$ and $\mu_{a0}=0.2\;{\mathrm{cm}}^{-1}$.^38^ Assuming constant optical properties in the tissue constitutes a first order approximation to the true fluence, which would require either prior knowledge or joint reconstruction of the scattering and absorption coefficients.

**Fig. 4:**
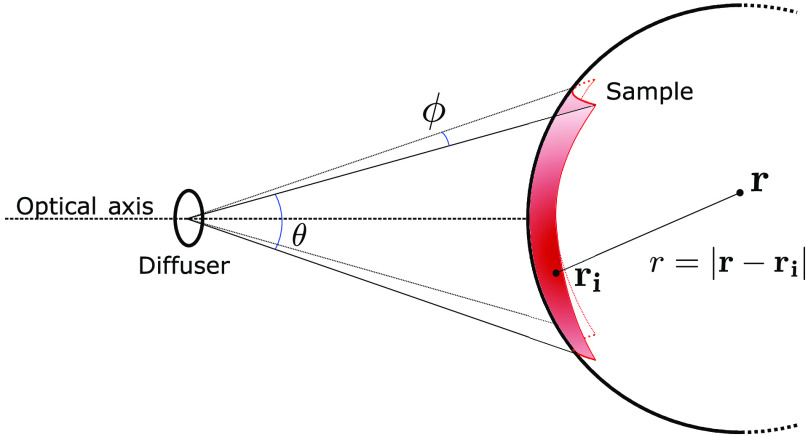
The coordinate system for the illumination system, indicating the divergence angles of the beam and the intersection with the sample surface.

The geometry of the illuminator and the resultant fluence at the sample surface is shown in Fig. 4. For a given position in the sample, r, the fluence can be computed by integrating Eq. (9) over values of $r_{i}$ on B, the section of the dome surface bounded by the angles θ and ϕ corresponding to the two diverging axes of the fan-shaped beam:

$$\Psi (r)=\iint_{B}\frac{1}{4\pi Dr}\;\exp(-\mu_{\mathrm{eff}}r)d\theta \;d\phi .$$ (11)

### 2.5 Reconstruction Implementation

**Fig. 5:**
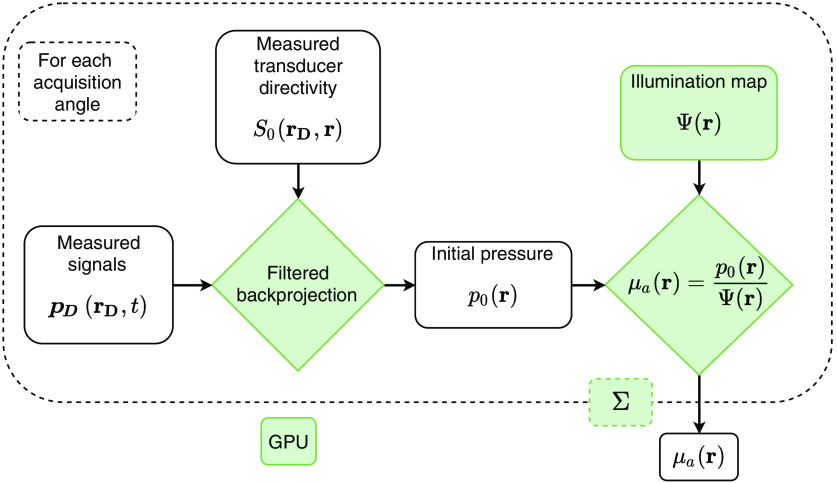
A schematic outline of our reconstruction scheme. Green boxes indicate operations computed on GPUs.

Figure 5 shows a summary of our reconstruction scheme. We first filter and back-project the measured signals to recover the initial pressure, taking into account the spatial sensitivity of the transducer array. We then compute the illumination map and use it to normalize the initial pressure, recovering the optical absorption map for the current illuminator position. It is important to reiterate that, since the illuminator is fixed relative to the transducer and not the sample, Ψ(r) must be recalculated for each acquisition angle. By applying this method for each angle, and combining the results, the optical absorption coefficient in the entire 3D tissue volume can be reconstructed. This accumulation over acquisition angles means that the final estimate of $\mu_{a}$ cannot be linearly separated into Ψ and $p_{0}$ as would be the case with systems employing stationary illumination.

For all results in the present study, we collected RF data at 200 planes with a constant angular spacing of 1.8°. We structured our GPU reconstruction into a number of blocks equal to the number of image voxels, i=1,⋯,N, with each 384-element acquisition plane divided into 384 threads. This was performed sequentially for each of the planes, with the results being accumulated in GPU memory.

We computed the illumination map given by Eq. (11) at each of the N image voxels by dividing the surface B into small patches and summing their contributions to the fluence within the sample. These contributions are independent and can thus be computed in parallel on a GPU. For the present study, we divided B into 200 patches, each of which was computed in a separate GPU thread, resulting in a 200× reduction in computation time.

Reconstruction was implemented in Python, with the following computationally-intensive portions implemented in CUDA: back-projection, filtering, SVD denoising, and calculation of the illumination map Ψ(r). Reconstruction was performed on a 64-bit Windows 10 PC with 16 GB of memory, an Intel Core i7-7700 processor (Intel Corporation, Santa Clara, California), and a GeForce GTX 1060 3GB GPU (Nvidia Corporation, Santa Clara, California).

All reconstructions in this paper have a resolution of 0.5 mm and assume a constant acoustic velocity of $1500\;m\;s^{-1}$. To calculate the illumination profile using Eqs. (10) and (11), we used the scattering and absorptive attenuation coefficients of milk at 700 nm^39^—$\mu_{s}=30\;{\mathrm{cm}}^{-1}$ and $\mu_{a0}=0.015\;{\mathrm{cm}}^{-1}$ for the calibration phantom and of human breast tissue at 700 nm^38^—$\mu_{s}=10.0\;{\mathrm{cm}}^{-1}$ and $\mu_{a0}=0.2\;{\mathrm{cm}}^{-1}$ for the chicken breast phantom.

### 2.6 Phantoms

To provide a robust and reproducible way to test our entire imaging system and reconstruction scheme, we designed and built a modular photoacoustic phantom.

**Fig. 6:**
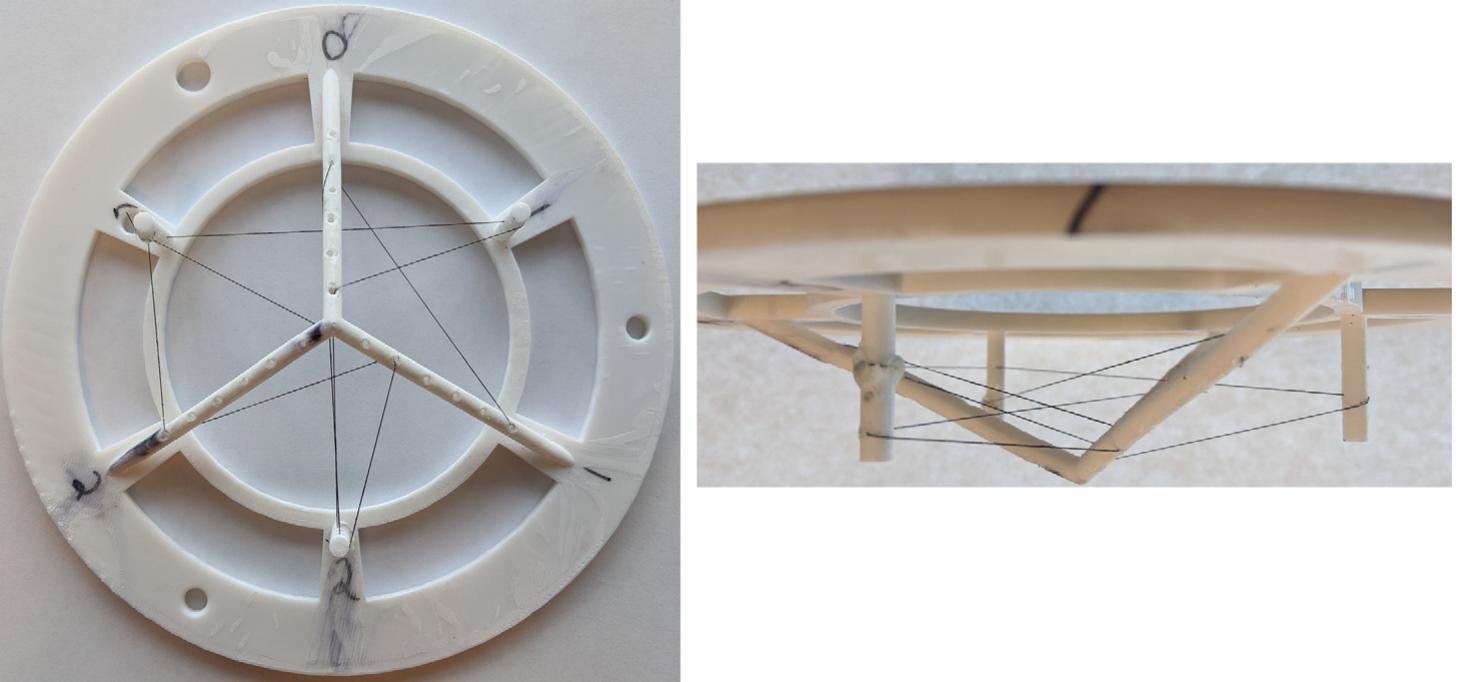
Images of the calibration phantom used in this study. The white template is 3D printed plastic, and black painted fishing line serves as a photoacoustic source. The circular portion of the template has an outer diameter of 19 cm.

As an absorber, we used 0.25-mm diameter monofilament fishing line, coated with black spray paint. This provided a strong photoacoustic signal, and the small diameter served as a test of the system resolution. To suspend the fishing line in the imaging volume, we designed and 3D printed a scaffold offering multiple mounting points between which the line was extended. The scaffold consisted of a base that precisely aligns the phantom with the imaging volume and several cylindrical columns with regularly-spaced holes through which the fishing line was threaded. This scaffold is shown in Fig. 6. As shown in Fig. 6, the fishing line was threaded through multiple points, providing a complex inclusion geometry that is nonetheless simple to describe and reproduce. For imaging the calibration phantom, the ABUS dome was filled with a 50/50 mixture of water and milk to provide optical scattering. To account for this mixture, we scaled the scattering and absorptive attenuation coefficients in our reconstruction by a factor of two, giving $\mu_{s}=15\;{\mathrm{cm}}^{-1}$ and $\mu_{a0}=0.0075\;{\mathrm{cm}}^{-1}$.

An additional advantage to using a 3D printed template is that we can easily use the design file to generate the inclusion geometry for use in simulations and validation of our reconstructed data.

To test our system in a more highly-scattering medium, we imaged a piece of 0.5-mm diameter pencil graphite embedded 2-cm deep in a piece of chicken breast tissue.

## 3 Results

### 3.1 SVD Denoising

**Fig. 7:**
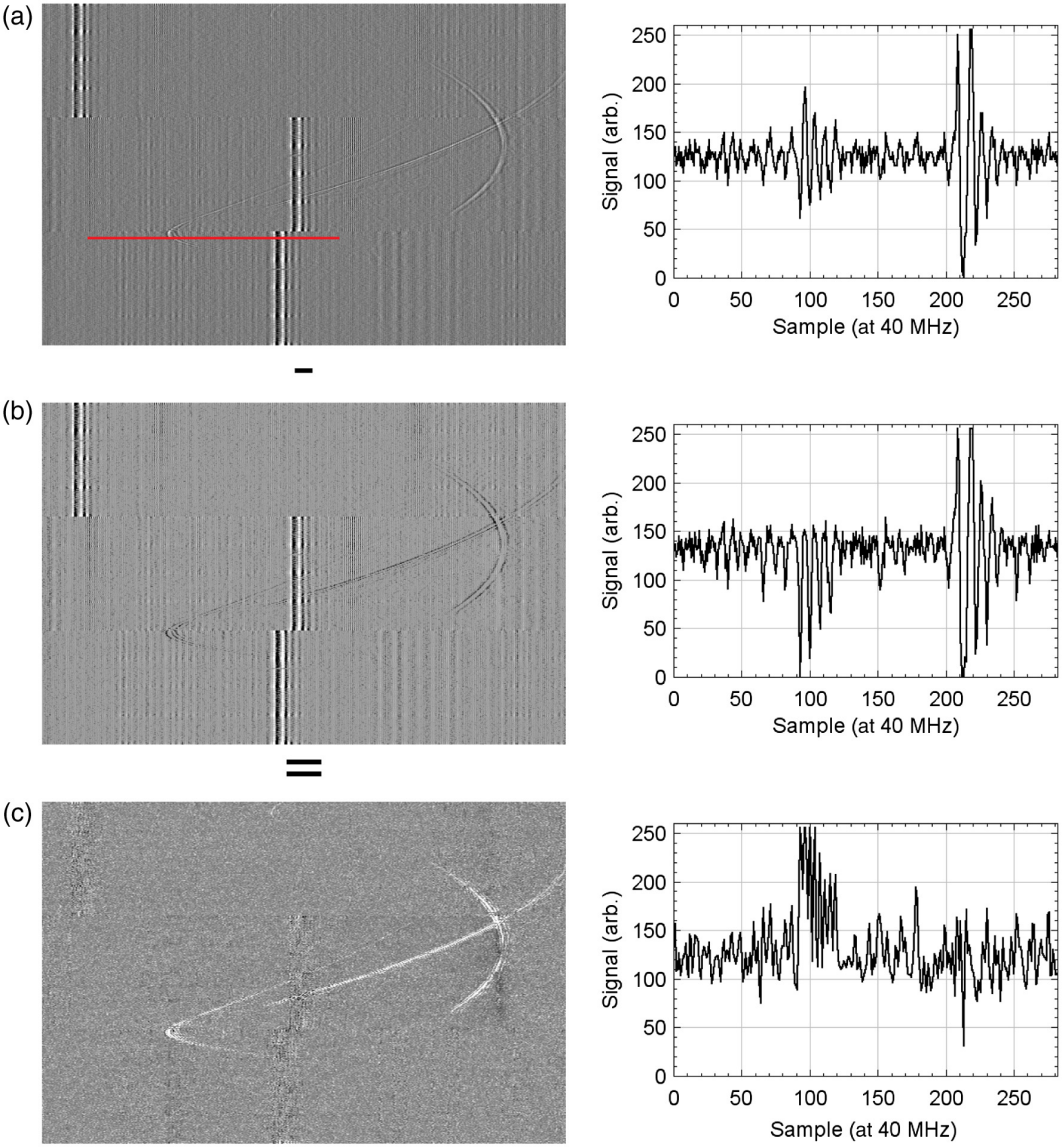
The effect of SVD denoising on the RF data. Right panels show the signal along the red line for each of the three images. Noise signal corresponds to the first 10 singular values. (a) The raw data without processing. (b) The isolated noise. (c) The subtraction of (a) and (b).

Figure 7 shows an example of the effect of the denoising method described in Sec. 2.3 on representative RF data. For our data, discarding the first 10 singular values produced the best balance between removing noise and maintaining signal, as characterized by measuring the SNR for an A-line containing a known inclusion signal.

### 3.2 Transducer Directivity

We measured the spatial sensitivity of the ABUS transducer, $S_{0}(\theta_{L},\theta_{E})$, by attaching a 2 mm long, 0.7 mm diameter graphite rod on a piece of fishing line to a 3-axis translation stage such that it was suspended in the water-filled ABUS dome. We coupled our laser system into the optical fiber as described in Sec. 2.2 and mounted the output end on the translation stage, directed toward the graphite inclusion. This ensured relatively constant illumination as the inclusion was translated. Photoacoustic data was acquired at a total of 200 points, over 1 cm in the elevational direction and 1.6 cm in the axial direction. The lateral position was fixed near the midpoint of the transducer since there are sufficient elements to constitute a range of lateral positions.

**Fig. 8:**
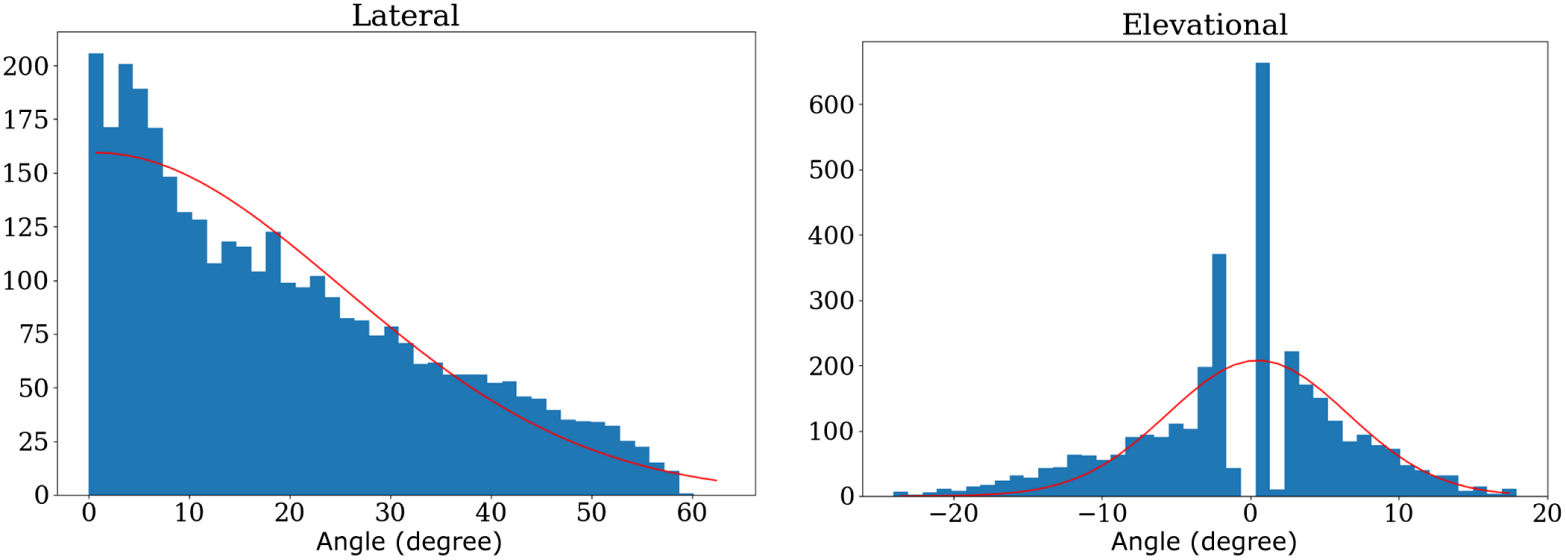
The sensitivity of the SonixEmbrace transducer elements as a function of the elevational and lateral angle between the element normal and the imaging point. Red lines indicate Gaussian fit.

We observed a Gaussian dependence of the signal intensity on the angle between the transducer normal and the inclusion position, both in the lateral and elevational directions. The measured standard deviations of these distributions, $\sigma_{L}$ and $\sigma_{E}$, corresponding to the lateral and elevational angles $\theta_{L}$ and $\theta_{E}$ was 18.5° and 6.2°, respectively. These data and the associated fits are included in Fig. 8.

### 3.3 Combined Imaging

**Fig. 9:**
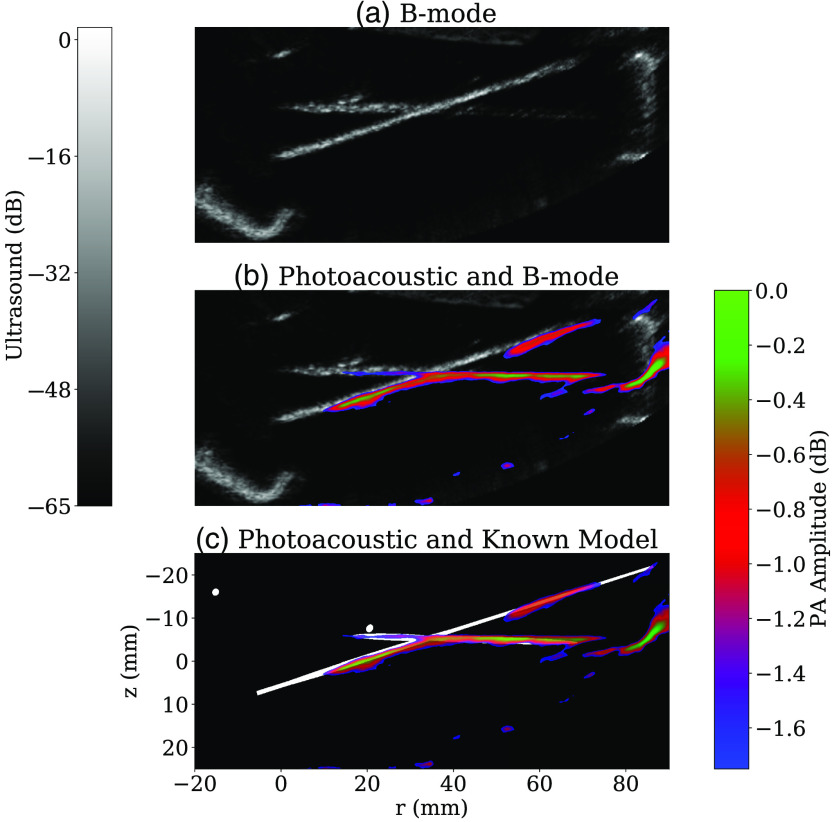
B-mode and photoacoustic data for a representative plane of the calibration phantom, indicating registration with modeled inclusion positions derived from the wire phantom design file.

Figure 9 shows representative in-plane images of the calibration phantom, demonstrating registered B-mode and photoacoustic imaging. For clarity of display, the photoacoustic data were thresholded such that only pixels with amplitudes in the top 10% of the image histogram were visible. The full dynamic range of the non-thresholded photoacoustic image was 10 dB. Figure 9(c) shows the model inclusion data derived from the design file for the 3D printed wire phantom, which further confirms that the designed structure of the phantom matches what we see when imaging. It is worth noting that the diameter of the inclusions in the model data in Fig. 9(c) are exaggerated 10× to be more easily visible. There are two circular features in the model data, corresponding to wires oriented normal to the imaging plane and therefore generating photoacoustic signals too weak to be above the display threshold. We used two measures to quantify the agreement between any two of these registered modalities. The first was the root-mean-squared error (RMSE), defined as

$$\mathrm{RMSE}=\sqrt{\frac{1}{N}\overset{N}{\underset{i=1}{\sum }}{\left(A_{i}-B_{i}\right)}^{2}},$$ (12)

where $A_{i}$ and $B_{i}$ are the value of the i’th pixel in the two images and N is the number of pixels in the image. The second metric used was the structural similarity index (SSIM) defined by Wang et al.^40^ We used the Python implementation of this function from the scikit-image package.^41^ Support structures from the phantom template (see Fig. 6) visible in the bottom left corner and far right side of the B-mode image were cropped out before performing these measurements. These comparisons are summarized in Table 1.

**Table 1:** Registration errors between imaging modalities.

	B-mode versus model	PA versus model	B-mode versus PA
RMSE	2.66	2.33	3.31
SSIM	0.48	0.78	0.40

### 3.4 Illuminator Spatial Profile

**Fig. 10:**
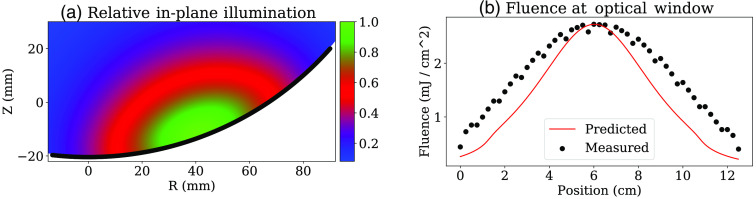
Optical fluence for one acquisition plane. (a) The two-dimensional structure of the illumination, relative to the transducer face, which is shown in black. (b) The measured fluence at the sample side of the optical window in black, compared with its predicted value, as a function of the position along the arc of the window.

Figure 10 shows the calculated illumination structure within the sample, as per Eq. (11). We also measured the fluence at the sample side of the optical window to estimate the overall losses in our fiber coupling and diffusing system. The measured data are shown in Fig. 10(b), alongside the modeled fluence for the same location. The broader shape of the measured data is due to the 1-cm aperture of the power meter used. Integrating the fluence over the entire window indicates a total energy per pulse of around 35 mJ, down from 100 mJ at the OPO output. If this same laser pulse were collimated to the size of the dome (surface area $250\;{\mathrm{cm}}^{-2}$), the average fluence at the tissue surface would be $0.14\;\mathrm{mJ}\;{\mathrm{cm}}^{-2}$.

### 3.5 Reconstruction Comparison

**Fig. 11:**
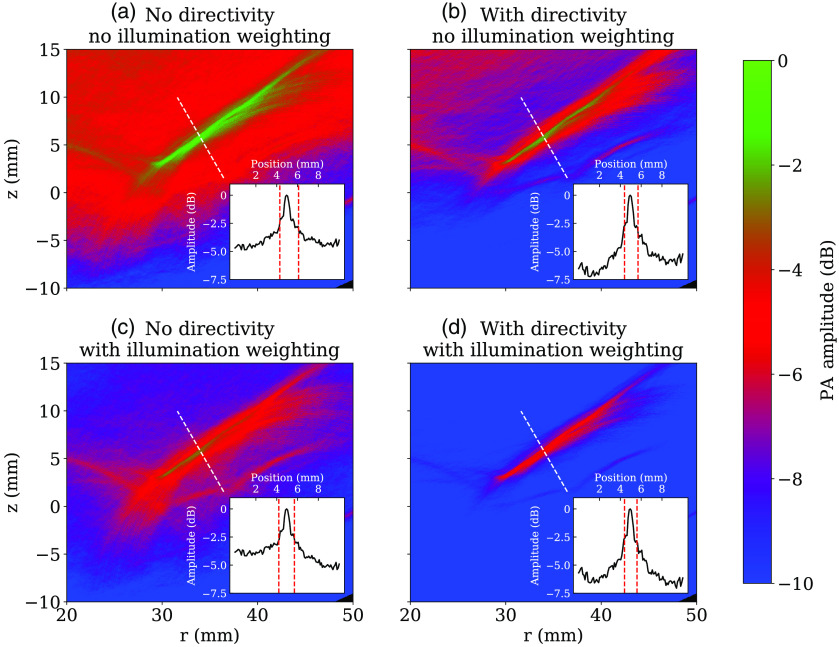
Comparison of reconstruction methods applied to chicken breast phantom. Insets show the PA amplitude along the dashed white line, normalized to the same dynamic range across all four images, with vertical dashed red lines indicating the FWHM of the peak. Corresponding measurements are summarized in Table 2.

To test our reconstruction scheme, we reconstructed our measured data both with and without accounting for transducer directivity and non-uniform illumination. We then measured SNR of a known inclusion for each method. We measured SNR here by extracting the photoacoustic amplitude along a line perpendicular to the long axis of the inclusion. We found the maximum of this signal and defined the “peak” as the region where the amplitude is greater than 50% of this maximum. We then defined the SNR as the mean amplitude of the signal in this region divided by the standard deviation of the amplitude over the entire image.^42^ The width of this peak region is the full width at half maximum (FWHM) and provides a measure of the resolving power of the system. Figure 11 shows an example of this measurement from the chicken breast phantom. A comparison of the measured values is given in Table 2. Figures 11(a)–(d) were scaled to the same dynamic range for display to make qualitative differences in the images clearer.

**Table 2:** Comparison of reconstruction methods applied to chicken breast phantom (see Fig. 11).

	No directivity	Directivity
No illumination compensation	SNR: 6.3 dB, FWHM: 1.8 mm	SNR: 7.1 dB, FWHM: 1.3 mm
Illumination compensation	SNR: 7.3 dB, FWHM: 1.5 mm	SNR: 8.0 dB, FWHM: 1.2 mm

**Fig. 12:**
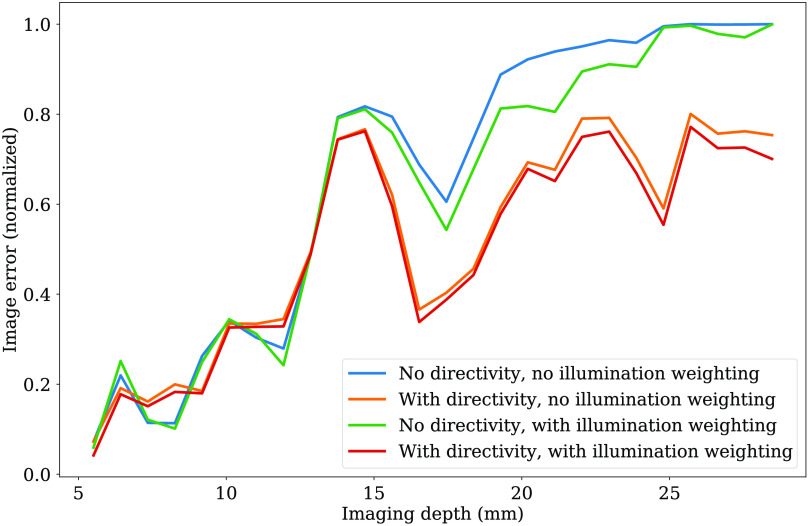
Image error as a function of imaging depth, compared for reconstruction schemes with and without transducer directivity and/or non-uniform illumination compensation.

**Fig. 13:**
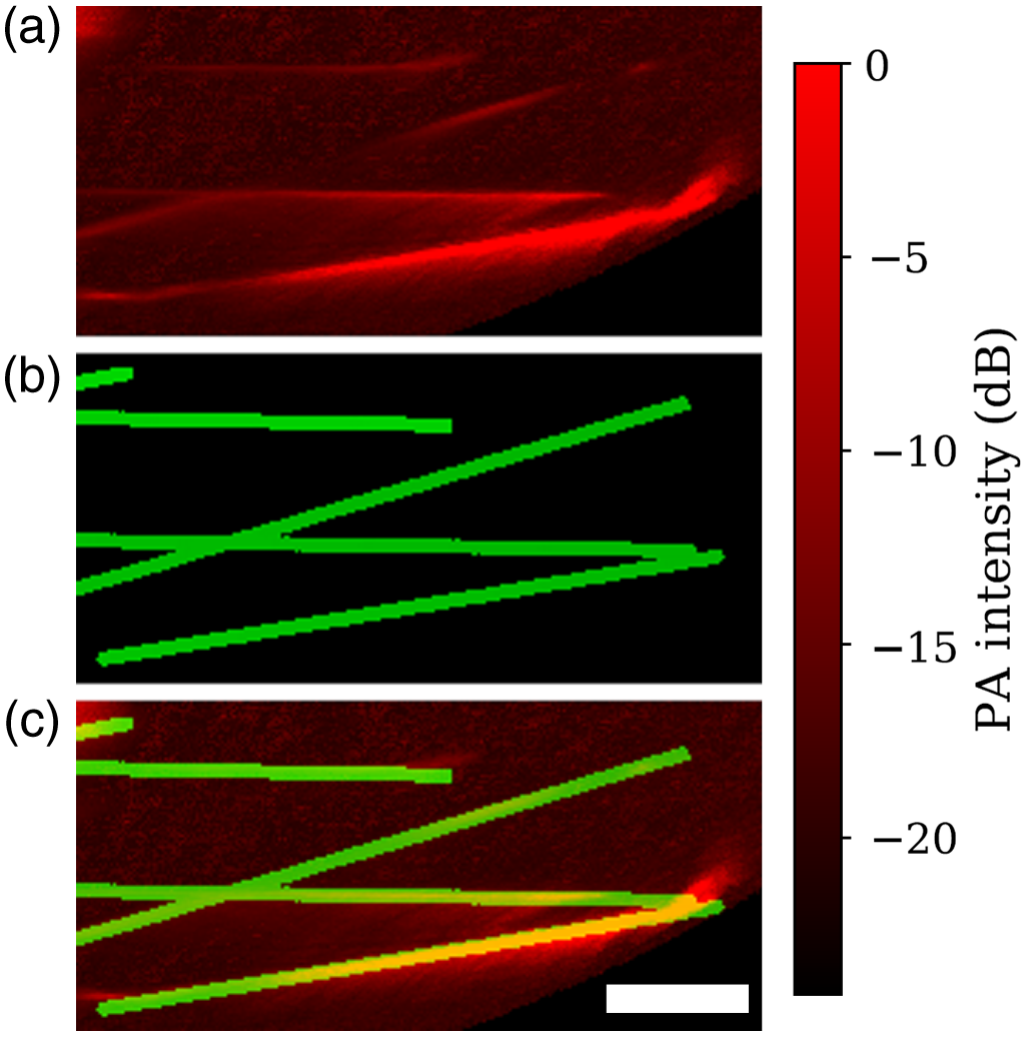
Maximum intensity projections of a tomographic reconstruction of the calibration phantom. (a) The data reconstructed with directivity and illumination compensation; (b) the model data; and (c) an overlay of the two. White scale bar indicates 1 cm.

To further quantify the performance of our reconstruction scheme, we compared the image error in this reconstruction as a function of imaging depth in our calibration phantom. Using the known model for the inclusions, $M_{i}$, we define image error as

$$\mathrm{ERR}=\frac{\left|A_{i}-M_{i}\right|}{\left|M_{i}\right|},$$ (13)

where $A_{i}$ is the reconstructed absorption value of the i’th voxel and |⋯| is the two-norm. We performed this measurement both with and without accounting for the directivity of the transducer and with and without accounting for the non-uniformity of the illumination. To measure the depth dependence, we divided the imaging volume into 50 equal-width spherical shells, each of which corresponds to a particular distance from the transducer surface. These data are summarized in Fig. 12. Figure 13 shows maximum intensity projections of a tomographic reconstruction of our calibration phantom, alongside the model data predicted from our design files. We note a broader dynamic range displayed in this data since the calibration phantom was imaged in a medium with lower light attenuation than the chicken breast tissue.

## 4 Discussion

Our combined B-mode and photoacoustic imaging results, as shown in Fig. 9, show qualitatively a strong agreement between both modalities and the model data, indicating no significant errors between the designed phantom template and the final image data. This reinforces a primary advantage of the ABUS as a multi-modal imaging platform, as no registration procedure was required between these data. As indicated by the registration metrics in Table 1, we see that the photoacoustic data are in closer agreement with the model, evidenced by a lower RMS error, and a higher structural similarity value. We expect that this is likely due to the photoacoustic data being similarly sparse and of high contrast, in comparison with the B-mode data.

Comparing our imaging results in chicken breast tissue, we see that our reconstruction method offers a significant improvement in both the qualitative appearance of the images in Fig. 11 and the quantitative improvement to the SNR values and resolution of an inclusion at a 2-cm imaging depth, as summarized in Table 2. We see that including either transducer directivity or illumination compensation increases SNR and improves resolution, whereas the best results are achieved when both are included. This measured SNR of 8 dB is comparable to the results of Wray et al,^43^ who achieved SNR values of 6.1 and 9.2 dB at depths of 1.5 and 1.8 cm, respectively, in human muscular tissue. We note that, in that study, the authors had a similar constraint of a single laser pulse per acquisition, but they used a transducer optimized for photoacoustic imaging. As such they were able to attain a much higher resolution of 255  μm, compared with our resolution of 1.2 mm.

Our calibration phantom image error shown in Fig. 12 shows that, at imaging depths >1.5  cm, both directivity and illumination compensation offer an improvement over the standard reconstruction. The effect of directivity is more significant, but the combination of the two consistently produces the smallest error. Adding both compensation terms improves the image error by an average of 18% over all imaging depths and by an average of 31% over imaging depths >1.5  cm. It is to be expected that our method would offer the greatest improvement at larger imaging depths since that is where the actual fluence will differ most from the assumption of uniform illumination.

We note that, since the illumination map Ψ must be computed for each acquisition angle, significant computation time is added to the reconstruction. For a tomographic reconstruction of the entire ABUS volume from 200 transducer planes, adding non-uniform illumination compensation increases the computation time from ∼5  min to ∼1  h.

Our calibration phantom design has the advantage that its physical description is fully determined by the attachment points of the wires in the 3D printed structure, which are known from the computer-aided design. While a vascular structure mimicking actual breast images, as presented by Schoustra et al,^29^ for example, would have been more relevant to the ABUS, uncertainty in the inclusion locations would have made the computation of metrics such as the image error much more difficult. Our next step is to acquire real images from healthy volunteers as performed in that study.

## 5 Conclusions

We developed an illumination scheme for PAT that can be implemented in cases in which there is insufficient space to illuminate the entire surface of the breast, as is the case with the SonixEmbrace ABUS. This illuminator can attain a fluence of up to $2.5\;\mathrm{mJ}\;{\mathrm{cm}}^{-2}$ at the tissue surface. We demonstrated the use of this PAT system to image the 0.25-mm features of our calibration phantom at depths of up to 3 cm in an optically scattering background.

We also devised a reconstruction scheme that can account for this non-uniform illumination, attaining a 25% increase in SNR at 2 cm in chicken breast tissue when compared with a reconstruction scheme that assumes spatially uniform illumination. We quantified the improvement that this reconstruction scheme affords, such that we can apply it on a case-by-case basis depending on whether image fidelity or reconstruction time needs to be prioritized. Implementing this algorithm on a GPU provided a 200× speed improvement over the same algorithm when executed on a CPU, for an identical imaging configuration (number of acquisition planes, and image grid size).

There are several limitations of our system that would be barriers to clinical translation.

1. Relatively slow scan time of 20 min. This is primarily due to the slow data transfer rate of the SonixDAQ module. To mitigate this, we are migrating to a Verasonics Vantage system (Verasonics, Inc, Kirkland, Washington) and exploring upgrades to our laser system to improve on our current pulse repetition rate of 10 Hz. We also currently stop the motor for each acquisition, but we intend to explore the possibility of continuous scanning.
2. Long reconstruction time. For this study, we reconstructed the entire ABUS volume; however, in a clinical application, we would likely be imaging regions of interest or 2D planes during the exam, while the entire 3D tomographic data would be reconstructed offline for later review by a radiologist, comparable to the workflow for modalities such as mammography or MRI. Nonetheless, we are exploring optimizations to our algorithms and moving to cluster-based computation for further improvement.
3. Persistent noise bands in the RF data. These are largely due to the relatively low amplitude of our PA signals. We are exploring further pre-processing techniques for reducing this noise, but the most promising path forward is to increase the fluence in the tissue by adding a second illuminator. We are also developing a regularized reconstruction scheme to further mitigate noise and reconstruction artifacts.

There are further improvements that could be made to our imaging results by more thoroughly characterizing the current array and moving to a reconstruction technique that fully accounts for the frequency-dependent spatial response of the transducer. We also intend to explore supplementing the SonixEmbrace transducer with a more broadband, purpose-built photoacoustic array. For the imaging of breast cancers undergoing angiogenesis, a center frequency of 2.25 MHz with sensitivity extending into the kHz range is ideal.^44^ Further, the inclusion of an elevational acoustic lens on the SonixEmbrace transducer limits the acceptance angle of the detection elements, leading to imaging artifacts and decreased sensitivity.^23^ Capacitive micromachined ultrasound transducers (CMUTs) have proven very promising in terms of frequency response and angular sensitivity,^45^^,^^46^ but they suffer from expensive and complex fabrication and are known to be delicate and therefore prone to degradation with use. Recent developments in fabrication techniques may alleviate or eliminate some of these issues, facilitating rapid prototyping of thin, flexible, transparent CMUT arrays.^47^^,^^48^
