# Supplementary material for: Integrating photoacoustic tomography into a multimodal automated breast ultrasound scanner
Source: J Biomed Opt. 2020 Nov 19;25(11):116010. doi: 10.1117/1.JBO.25.11.116010 (PMC7675066; doi:10.1117/1.JBO.25.11.116010)
Supplement: Supplementary file 1 [file JBO_025_116010_SD004.pdf]

Supplementary Information

Data denoising

Figure S1 shows a comparison of denoising methods applied to our raw radio frequency (RF) data, along with peak signal-to-noise ratio (PSNR) measurements for an A-line which passes through both a known photoacoustic signal and a noise band. We define PSNR here as the peak amplitude of the known signal (the green region in Figure S1) divided by the standard deviation of the entire A-line.

First, we use the spatio-temporal filtering method of Manduca *et al*,[33] with the filter oriented to remove vertical features. While this method did significantly reduce the prominence of the noise bands relative to the photoacoustic signals, as evidenced by the PSNR improvement, the bands are still clearly visible in the data. We expect this is due to these bands being non-uniform across different elements, resulting in significant frequency content passing the filter.

With the SVD-based method of Hill *et al*,[31] as described in the main text, we see that the noise bands are completely suppressed, and the PSNR is higher than that achieved with the directional filtering method.

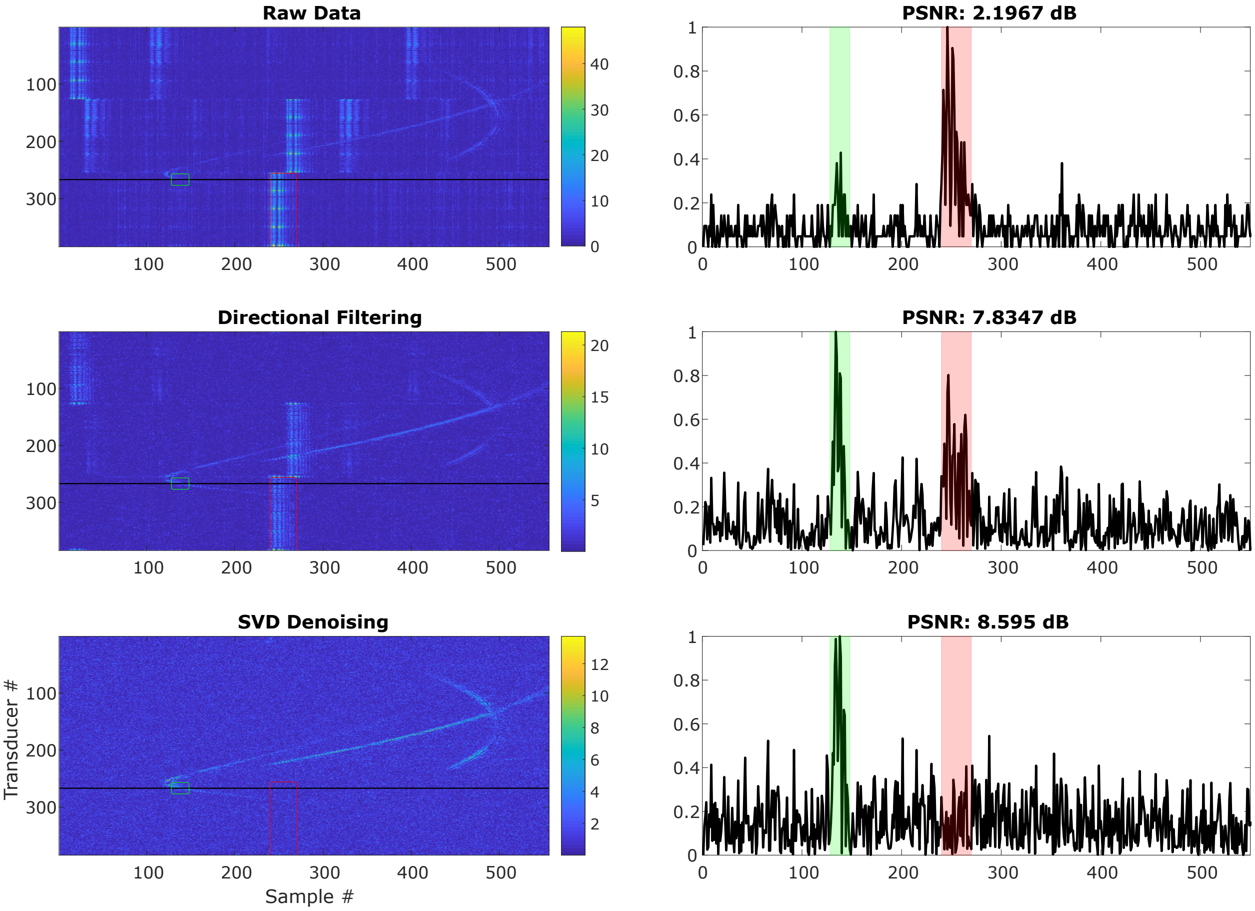

Fig S1 Comparison of data denoising methods. Images on the right show RF data for each transducer element. Plots on the right are the RF signal along the black line in the image plot, with actual photoacoustic signal highlighted in green and a representative noise band highlighted in red.
